# Temporary spanning plate across the elbow for complex fractures of the distal humerus

**DOI:** 10.1186/s12891-021-04764-x

**Published:** 2021-10-13

**Authors:** Ashraf N. Moharram, Mostafa Mahmoud, Ahmed Lymona, Ahmed Afifi, Mostafa Ezzat, Mohamed Abdel-Wahed

**Affiliations:** Kasr El-Ainy Faculty of Medicine, Cairo University, Cairo, Egypt

**Keywords:** Distal humerus, Comminuted articular fractures, Spanning plate, Elbow fractures

## Abstract

**Background:**

Open reduction internal fixation (ORIF) is the gold standard management of fractures of the distal humerus. Stable fixation to allow early mobilization is not always possible in cases with comminuted fracture patterns and bone loss, with a high failure rate. We propose augmentation of internal fixation in these unstable situations with a spanning plate across the elbow to protect the fixation construct temporarily until bone union.

**Methods:**

Eighteen patients with complex distal humeral fractures were managed with standard ORIF technique augmented with a temporary plate spanning across the elbow as an internal fixator. Cases included were either very distal, comminuted (6 cases) or insufficiency fractures (4 cases) or revision fixation cases (8 cases). The temporary spanning plate was removed as soon as signs of early radiographic union were detected.

**Results:**

Seventeen patients were available for final follow up at a mean 28.3 months. The spanning plate was removed after 3.4 months on average. At the final follow-up, the mean elbow total arc of motion was 86.3°. The mean Mayo Elbow Performance Score (MEPS) was 80, and the mean Quick Disabilities of the Arm, Shoulder and Hand (Q-DASH) score was 27.

**Conclusion:**

Spanning the elbow temporarily with a plate in adjunct to standard ORIF technique is both simple and effective in achieving fracture stability and union and minimizes failure rates after fixation of comminuted, very distal fractures, osteoporotic cases, or revision fixation cases with bone loss.

**Level of evidence:**

Level IV, Therapeutic study

## Background

Different treatment options have been described for management of comminuted and insufficiency fractures of the distal humerus. Nonsurgical treatment in the form of immobilization (bag of bone technique) delays rehabilitation and usually ends up with unstable movement through the nonunion site above a stiff elbow joint [1, 2]. It should be limited to morbid patients or when other treatment options are not available [3, 4]. Surgical options include open reduction internal fixation (ORIF), hinged or non-hinged external fixation, and elbow replacement. The advent of anatomic preshaped locked plates and elbow arthroplasty have changed the management of distal humerus fractures [1, 4, 5].

Despite the advances of anatomical locked plates that allowed more stable fixation of distal difficult fractures than the standard 3.5 plates, there are still certain fractures that cannot be rigidly fixed by these plates. Tenuous fracture fixation should be expected with osteoporosis, low fracture line, severely comminuted but reconstructable fractures, and revision fixation cases [6].

External fixation can be added in these cases; however, it is usually not well tolerated and carries the risk of pin tract infection and pin loosening [6–8]. Total elbow arthroplasty (TEA) or distal humeral hemiarthroplasty (DHH) is used for older patients with comminuted fractures where reliable internal fixation is impossible. Arthroplasty of the elbow has lifelong restrictions and revision rates are still high [9, 10].

**Fig. 1:**
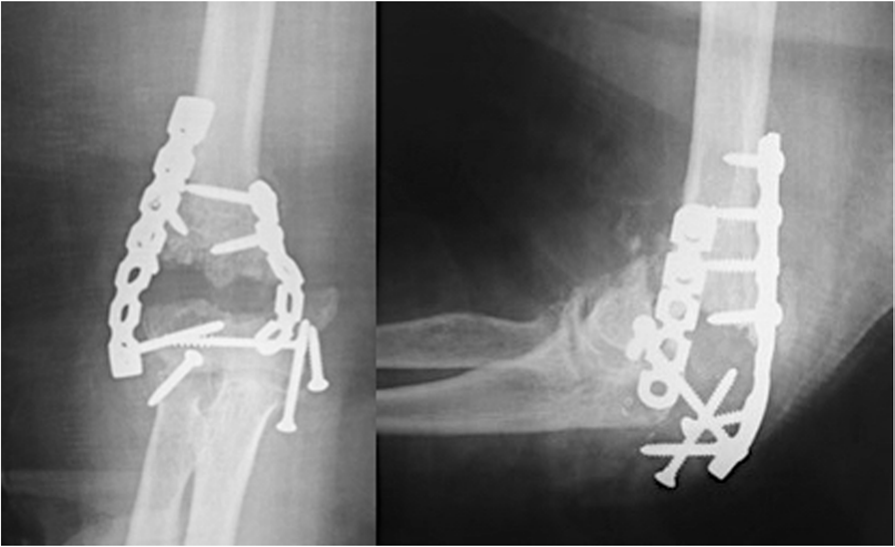
X-rays at presentation of a 69 years’ old lady (case #9) with low-laying fracture of the distal humerus that have failed a previous osteosynthesis

**Fig. 2:**
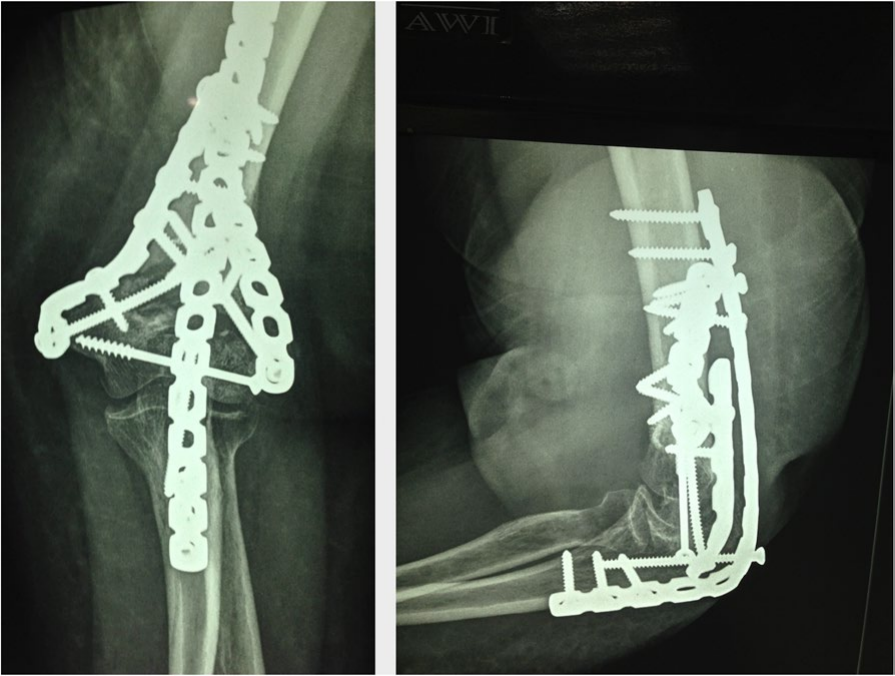
Postoperative x-rays of the forementioned patient (case #9) fixed with double columns plates and augmented with a temporary spanning elbow plate

**Fig. 3:**
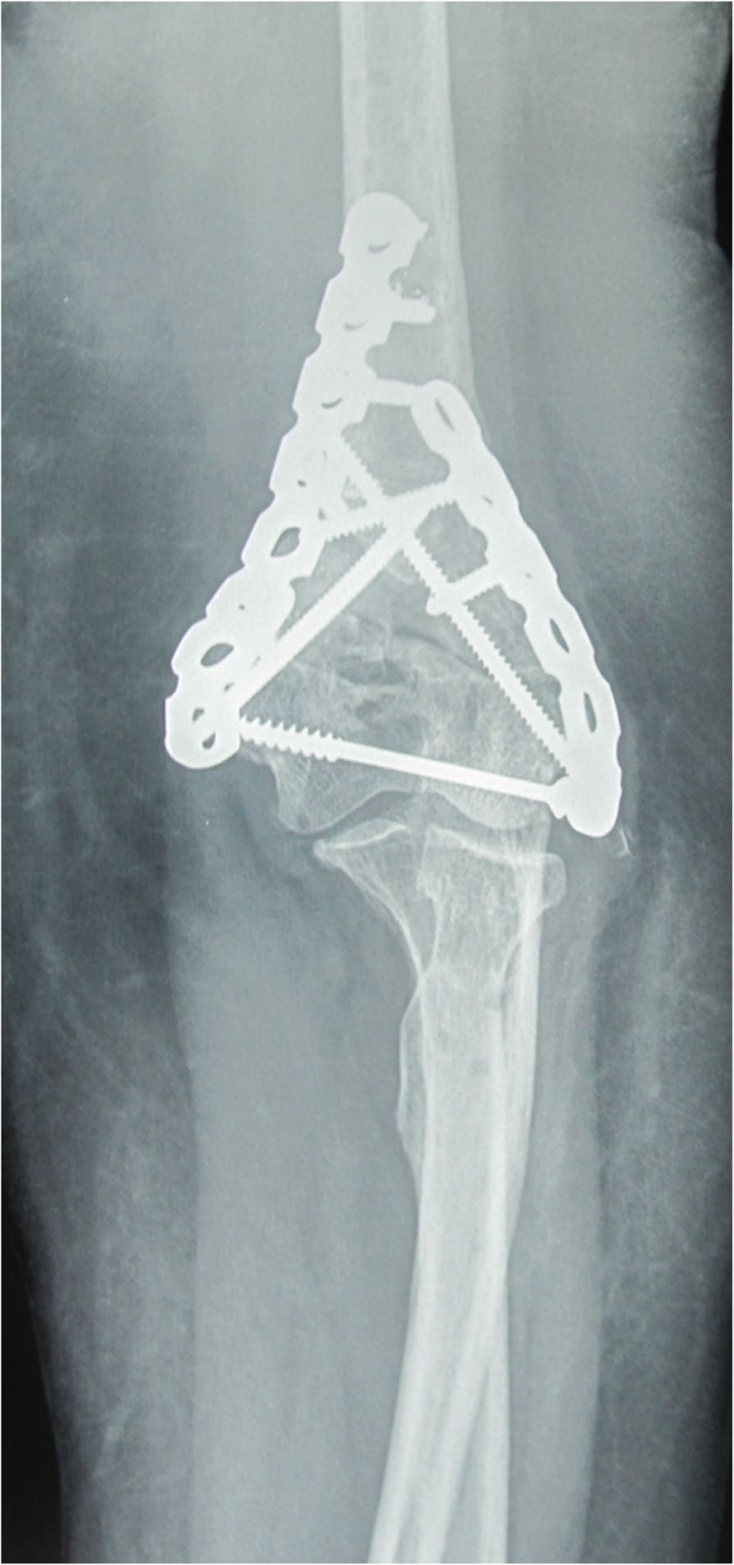
Same patient (case #9) after removal of the spanning plate 2.5 months later showing adequate union

In this series, we used the standard ORIF technique for distal humerus using locked plates, augmented with an ulno-humeral plate spanning across the elbow to add stability to the fixation construct in unreliable situations, temporarily until union (Figs. 1, 2 and 3).

## Methods

Between 2011 & 2019, all patients presented with complex distal humerus fracture were consented to have a staged procedure for management; composed of a first stage for standard ORIF augmented with a temporary ulno-humeral plate spanning across the elbow, and a second stage for removal of the temporary spanning plate after fracture consolidation. All patients were informed that the final decision to use the spanning plate is taken intra-operatively after fracture fixation when the fixation construct was judged to be not stable enough to allow early ROM, this was the situation with 18 patients who needed this technique and included (Table 1) very distal, comminuted (6 cases) or insufficiency (Osteoporotic) fractures (4 cases) or revision fixation cases (8 cases). The other patients were judged to have acceptable degree of stability with fracture fixation and therefore the step of applying the spanning plate was omitted and those patients weren’t enrolled in the study.

**Table 1:** Description of the included cases in the study

Mean age	49.8 years (range, 24–69)
**Sex (males: females)**	12:6 (66.6%:33.3%)
**Side (right: left)**	11:7 (61.1%: 38.9%)
**Dominant side involvement**	13 (72.2%)
**Types of the fractures**	
**• Acute fractures** *(AO/OTA classification)*	6 patients (33.3%) *(AO/OTA type 13C3.2 in 2 patients and type 13C3.3 in 4 patients.* 2 of these patients were neglected)
**• Insufficiency fractures (osteoporosis)**	4 patients (22.2%)
**• Failed previous fixation**	8 patients (44.4%)
**Types of the used spanning plates**	Nonlocking 4.5 mm narrow DCP’s (4 patients) (22.2%) Locking 4.5 mm narrow DCP’s (5 patients) (27.7%) Nonlocking 3.5 mm reconstruction plates (9 patients) (50%)
**Mean follow-up period**	28.3 months (range, 18–36)

The mean age of the 18 patients included in the study was 49.8 years. Patients were 12 males and 6 females. Cases with poor soft tissues, and potentially infected compound fractures were excluded. Deep infection was excluded in revision cases. Exclusion was extended to cases with unsalvageable articular surface. Two patients had ipsilateral limb fractures (Both bones forearm and midshaft humerus). Out of the 8 revision cases, half of them were operated only once before, while each of the other 4 underwent 2 separate fixation surgeries that ended up either with nonunion or loss of fixation before presentation. Two primary cases were neglected ununited cases at presentation (8 and 12 months).

### Technique

**Fig. 4:**
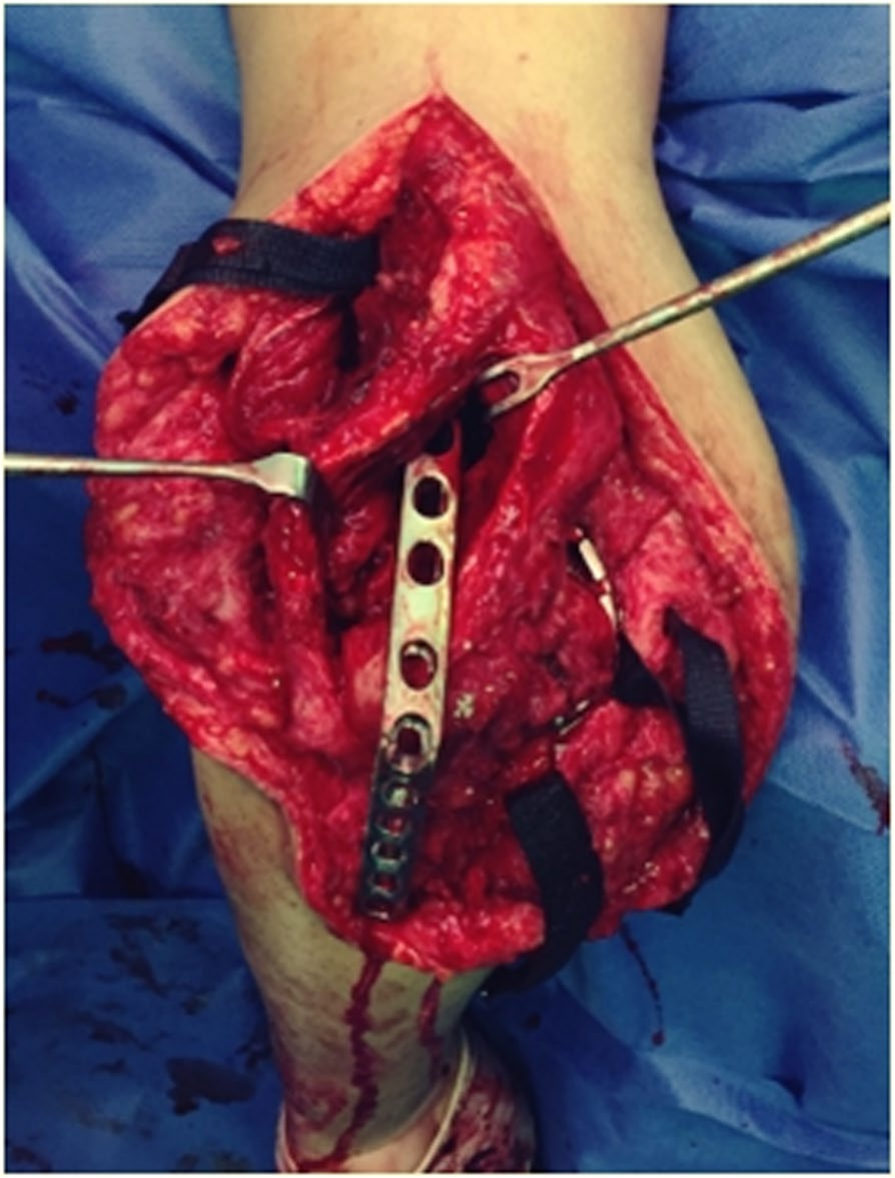
Triceps split (window) to apply the contoured spanning plate. In this patient (case #4) a non-locked DCP was used as a span plate. The triceps split window was relatively larger than usual and was the main window used for osteosynthesis with minimal paratricipital exposure

**Fig. 5:**
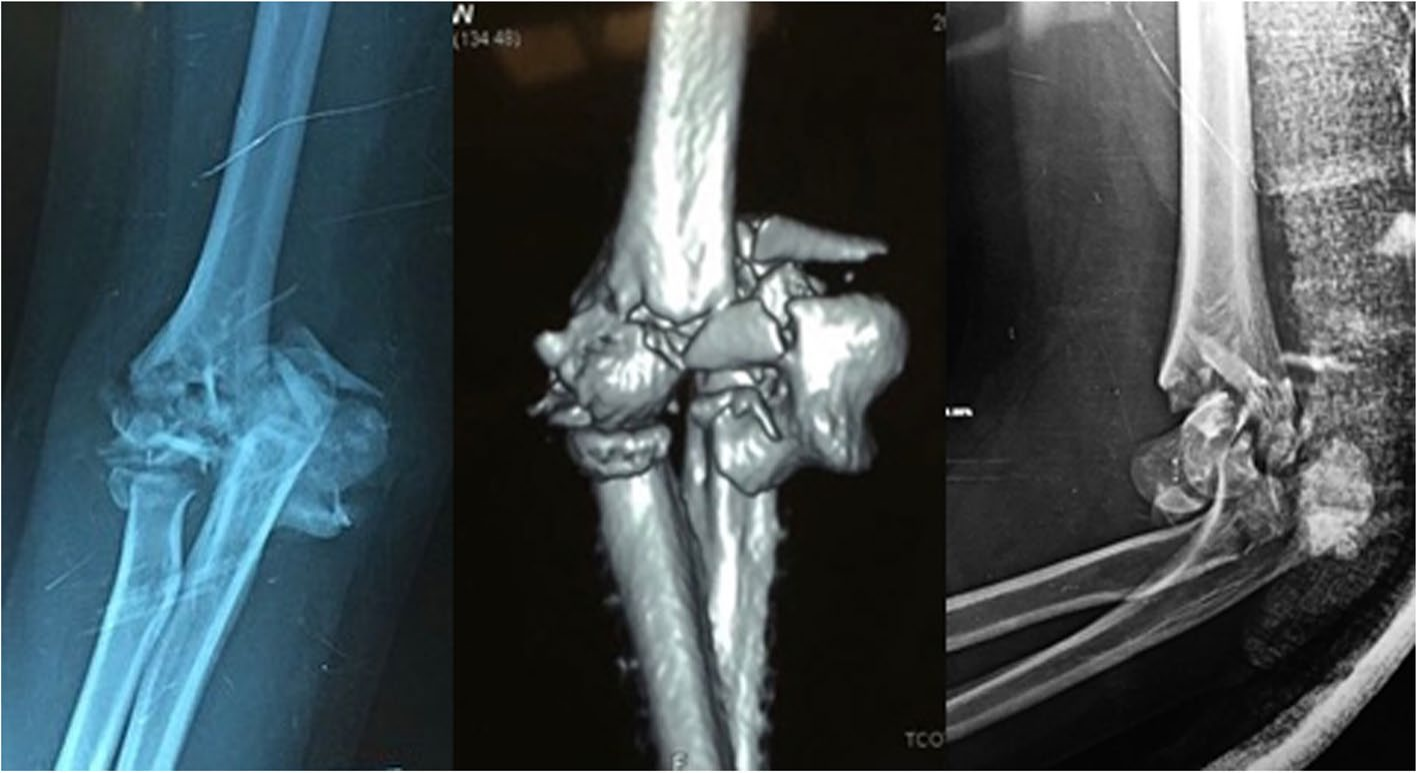
X-rays at presentation of a 19 years’ old female (case #14) with highly comminuted fracture of the distal humerus at the dominant hand

**Fig. 6:**
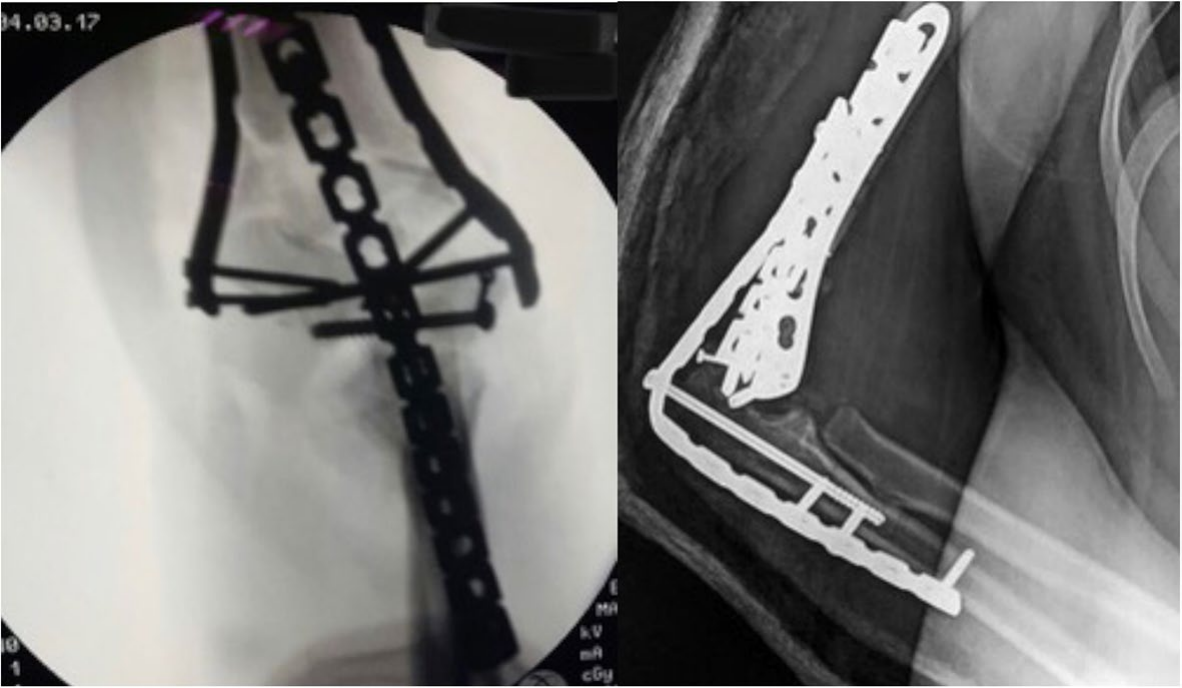
X-rays of the forementioned patient (case #14) fixed with preshaped column plate and augmented with a temporary spanning elbow plate. Olecranon osteotomy in this patient was fixed with an intramedullary screw inserted through the plate

**Fig. 7:**
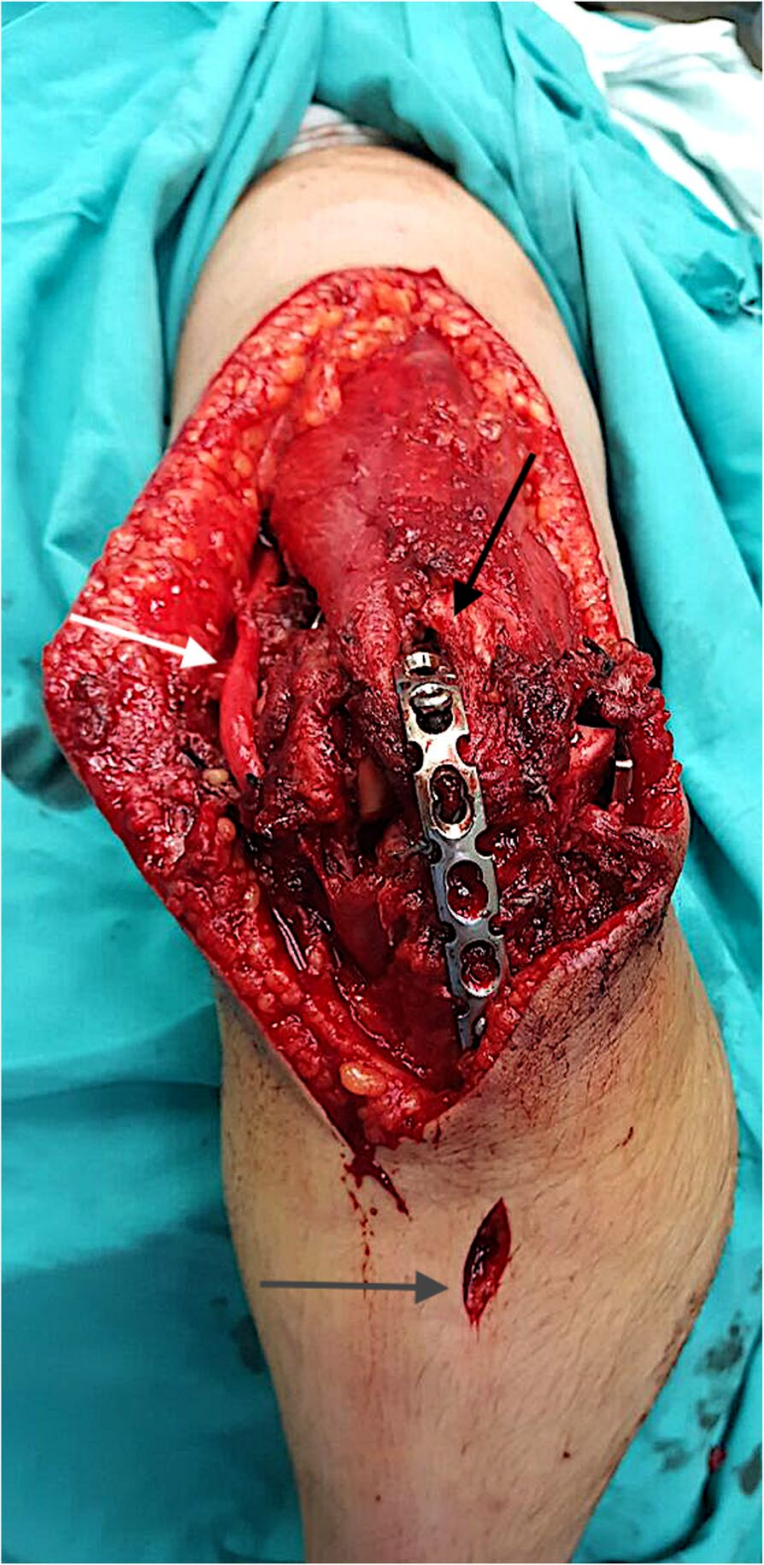
A reconstruction 3.5 plate was utilized as a spanning plate in this patient (case #14), inserted through a small tricipital window (black arrow) and an intramedullary screw is inserted through the plate to simultaneously fix the olecranon osteotomy. The fixation on the ulna side needed only a small window (grey arrow) to insert the screws. The ulnar nerve was released in situ (white arrow)

**Fig. 8:**
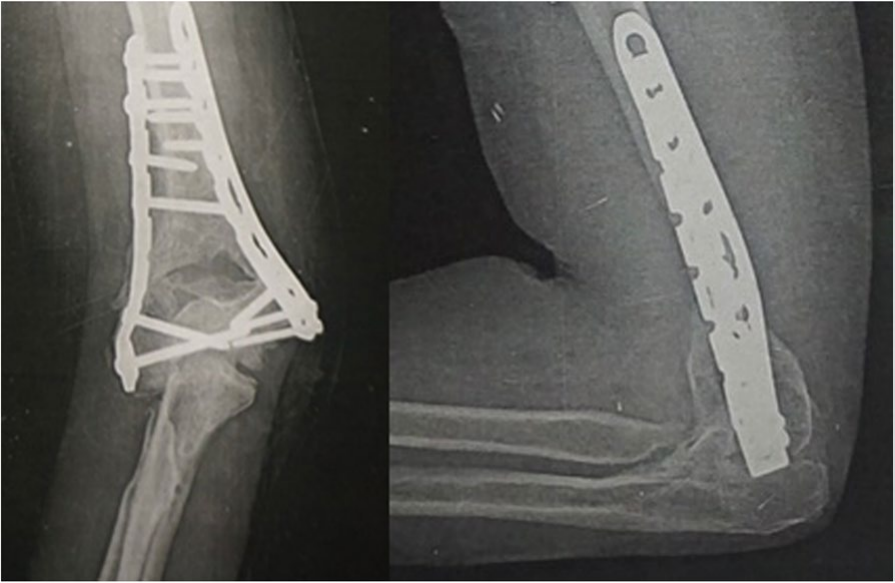
Follow up X-ray for the same patient (case #14) 9 months after spanning plate removal showing good union

After positioning at lateral decubitus; the fracture was exposed through posterior approach to the elbow and the ulnar nerve was isolated. The distal aspect of the humerus was exposed on both sides of the triceps. A small triceps splitting window was created just proximal to olecranon tip to visualize the articular surface and to allow the passage of the ulno-humeral plate deep to the muscle (Fig. 4). In cases with proximal extension of fracture line, radial nerve was identified and isolated. Fractures were then fixed using the standard AO principles. The orientation of plates applied to the humeral columns differed according to fracture configuration, with our preference to parallel plate rather than the 90/90 orientation. Assessment of construct stability was then checked clinically and fluoroscopically. A narrow, locked or non locked, LC-DCP or a reconstruction 3.5 plate was bent to cross the elbow in 90 to 100 degrees of flexion. The choice of the plate depended upon the relative bone size for each case, and the space available after plate fixation of the distal humeral columns. Plates were contoured to fit precisely the proximal ulna and the distal humerus. The spanning plate was fixed with a minimum of 3 screws on each side of the ulna and the humerus. Olecranon osteotomy was needed in 5 cases to adequately visualize and anatomically reduce the articular fracture. The osteotomy was fixed with an intramedullary screw either before application of the spanning plate (3 cases) or through the spanning plate (2 cases) (Figs. 5, 6, 7 and 8). Finally, the triceps split was then closed. Post-operatively, the elbow was splinted in an arm sling. Patients were encouraged to use their hands, wrists and to supinate and pronate their forearms as well as to move their shoulders.

**Fig. 9:**
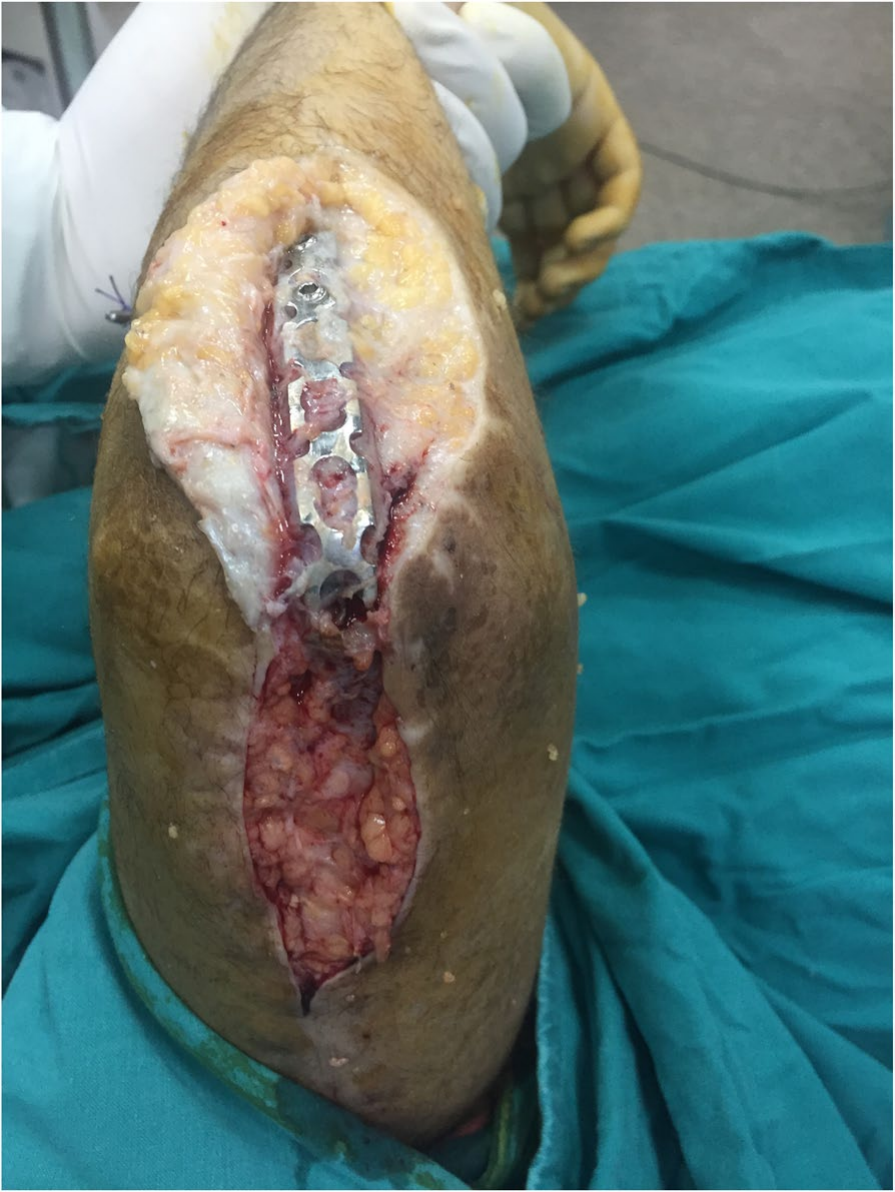
The same posterior approach was utilized to remove the spanning plate. In this patient (case #14) the spanning plate was removed after 3 months

**Fig. 10:**
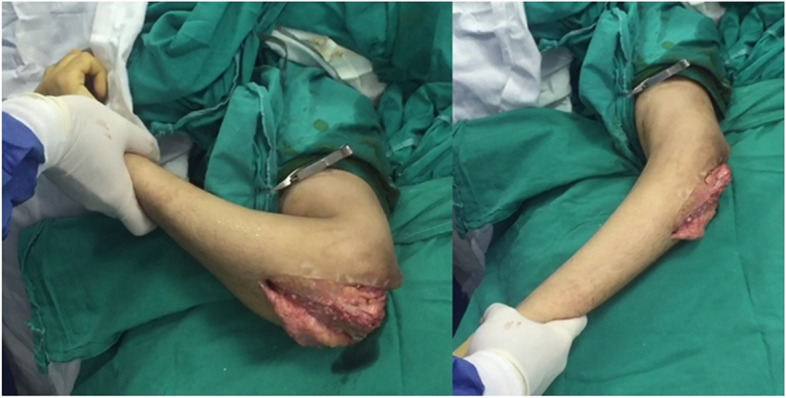
Gentle manipulation at time of removal of the spanning plate (case #14) with a functional range of motion achieved easily on table

The Ulno-humeral spanning fixation was removed as soon as evidence of fracture healing in the form of bone trabeculae crossing the fracture site appeared on X-rays. The intention to remove the plate typically between 2 and 3 months postoperatively was not possible in cases residing in remote areas. The same posterior incision was used (Fig. 9); triceps was split down to the plate. The elbow was gently manipulated through range of flexion and extension (Fig. 10). Implants stabilizing the original fracture were never removed in the same setting except for a prominent screws or wires. Patients were enrolled in a condensed course of active and active assisted exercises of elbow flexion & extension after the removal surgery. No load bearing restrictions were placed on the patients.

The primary outcome measure was the Mayo Elbow Performance Score (MEPS) [11]. Secondary outcome measures were fracture union, elbow flexion/extension arc, the Quick Disabilities of the Arm, Shoulder, and Hand (Q-DASH) score [11], and complications (e.g., infection, nonunion, or heterotrophic ossification). All outcome measures were recorded by an observer who was blinded to the procedure.

## Results

The mean follow-up period was 28.3 months, from the temporary spanning plate removal surgery (18 months minimum and 36 months maximum). One patient was lost to follow-up and 17 were available with full data at final enrollment. The temporary spanning plate was removed at an average of 3.8 months (ranging between 2.25 and 6 months). At the time of removal, all patients were united. The patient that removed the plate after 6 months had travel issues that delayed the second stage. Patients regained a satisfactory range of elbow movement of an average arc of 86.3° (range 65° - 130°). The extension lag ranged from 10° to 55° with a mean of 33°. Flexion recovery was better than extension and ranged from 110° to 140°. All patients had full range of supination and pronation.

At the final follow-up, the patients achieved a mean Mayo Elbow Performance Score (MEPS) of 80 (range 65–95), and a mean Quick Disabilities of the Arm, Shoulder and Hand (Q-DASH) score of 27 (range 13–43). This corresponds to a fair to excellent results; 3 fair, 9 good, and 5 excellent results. Complications included wound dehiscence in 1 patient after plate removal and radial nerve palsy in another patient. Wound dehiscence improved by daily dressing. Radial nerve palsy improved spontaneously after 5 months. A broken spanning plate without failure of the distal humeral fixation occurred at 3 months. This was removed with no need for additional procedures. No heterotopic ossification was observed. No patients developed ulnar neuropathy. Additional procedures in the form of removal of the remaining implants were done in 2 patients. One patient needed posterior elbow release and osteophyte excision to improve the extension range.

## Discussion

Reconstruction of the distal humerus following comminuted fractures remains a challenge even to the most experienced orthopedic surgeons. Open reduction and stable fixation is the aim, to start early mobilization [12, 13]. Through ORIF, these patients can return back to their activity without limitations on weight bearing. Stable fixation is not always possible in cases with low fractures close to the joint, the presence of metaphyseal comminution, articular fragmentation, and poor bone quality in osteoporotic patients and revision cases [6, 14]. In these cases, failure of achievement of stable internal fixation is common and is associated with high failure rates and poor outcome [15, 16].

TEA has been proposed as an alternative to overcome this difficulty. It offers immediate stability, early mobilization, and better short-term results in elderly low-demand patients. However, its main disadvantage is that patients undergoing TEA have lifelong activity restrictions and limitations on weight bearing. Moreover, the incidences of implant loosening and periprosthetic fracture are significant [10, 17]. Prasad et al. [18] showed that only 53% of non-rheumatoid patients who undergo TEA for a distal humerus fracture had implant survival of more than 10 years, and that 89.5% of patients demonstrated loosening of their prosthesis at this stage. Males had a higher incidence of loosening and wear; this may be attributed to the fact that the efficacy of TEA depends largely on long-term patient compliance to load restrictions leading to loosening of the implant. Therefore, TEA performs better in elderly patients with sedentary lifestyle.

DHH is another option that may be more durable than TEA as DHH has no ulnar component or polyethylene; therefore, theoretically the risk of component loosening is reduced. Despite the potential advantages of DHH over TEA, long-term follow-up results are still to be available. As for any other hemiarthroplasty, cartilage wear remains the most common challenge. Kawk et al. systematic review and meta-analysis showed that cartilage wear occurred in 39.1% of patients after DHH for distal humeral fractures [19]. It is to be noted that DHH usage in USA is off-label as it did not get a Food and Drug Administration (FDA) approval yet [20].

The concept of temporary spanning plate for periarticular fractures have been used to improve the results of standard ORIF techniques in wrist fractures, interphalangeal joints fractures and pelvic fractures. The expected role is to temporarily shield the mechanical loads across the fracture site. The dorsal wrist spanning plate in comminuted distal radius fractures is the one thoroughly studied and now is a standard technique for complex intraarticular and comminuted distal radius [21]. Spanning fixation for comminuted fractures of the small joints of the hand is emerging [22]. The hallmark of spanning fixation techniques is that it allows the restoration of the functional range of motion of the nearby joints through internal fixation with the addition of a temporary spanning plate that is taken off as soon as the fracture shows radiological signs of union. This preserves the native bone stock in fracture types otherwise deemed to need either replacement (as in case of elbow joint) or salvage arthrodesis (distal radius and small joints of the hand). Despite being a more economical option than TEA, there is an added need of another procedure to remove the spanning plate.

To the best of knowledge; No single study used spanning plates to temporarily protect fixation of elbow fractures. Two studies reported the use of spanning elbow plate but for cases of elbow instability. The technique of applying the temporary plate is similar. In Edelman’s series, 6 patients with a mean age of 52 years with terrible triad injury of the elbow were treated by temporary elbow bridge plate that was taken off between 4 and 6 weeks [23]. The other study is a case report of a subacute elbow dislocation with a good final range using temporary elbow bridge plate [24].

We claim that spanning plate augmentation of standard ORIF techniques of the distal humerus have the advantage of minimization of postoperative restrictions as well as revision surgeries. In our series, no fixation failure was encountered. Fair to excellent results were achieved in all patients with a mean arc of motion of 86.3° at a mean follow-up of 28.3 months. Our results are comparable to the results of the randomized controlled trial of McKee and his colleagues [25] in which they compared between internal fixation and primary TEA for 42 elderly patients with displaced intra-articular distal humeral fractures. In their study, the mean flexion-extension arc was 107° (range, 42°-145°) in the TEA group and 95° (range, 30°-140°) in the internal fixation group. Nevertheless, our study population had more difficult fractures and near half of them were revision cases (44%). The results of this series were also close to the results of DHH after distal humeral fractures. Schultzel et al. [26] followed-up 10 patients with DHH for an average of 73.2 months and showed maintained improvements in MEPS (mean, 89.23; range, 75–100) and DASH scores (mean, 33.71; range, 11.2–55.1).

In our series, immobilization of the elbow with the spanning plate offered the adequate mechanical environment for proper bone healing with moderate impact on the final range. Patients achieved functional flexion-extension range and there was no compromise of the pronosupination range in any case. This may be one of the advantages of internal splinting of the elbow with the spanning plate rather than external splinting with slab or cast. Pronosupination exercises started immediately postoperative in these cases. Immobilization of the forearm for a short period may be needed in fractures distal to the elbow with minimal impact on the final range [27, 28].

The limitation of the current study is the absence of a control group and the heterogeneity of the study population. Implants used were not unified in all cases. Early in the study, osteosynthesis was achieved using reconstruction plates contoured intraoperative according to the shape of the distal humerus. Later in the study the pre-contoured plates were available and this reduced the operative time but did not omit the need for the added stability offered by the spanning plate in tenuous situations. The exact timing of removal of the spanning plate is another limitation. We proceeded for this stage based on early radiological evidence of bone formation, this was expected between 2 and 3 months postoperatively. There is no accurate objective measure to precisely decide the timing of plate removal, but we depended on the support afforded by the columnar plates till fracture union.

Finally, we admit that the triceps muscle and tendon have been exposed to significant surgical trauma from the index procedure and the subsequent plate removal; however this impacted little on the elbow extension power.

## Conclusion

We added a simple technique to the armamentarium available for management of complex distal humeral fractures. Results achieved are comparable to elbow replacement, with the added advantages of preservation of native bone stock and avoidance of lifelong restriction of activity following replacement surgery. We recommend spanning plate augmentation of standard ORIF techniques in osteoporotic, comminuted but reconstructable, low fracture line or revision fixation cases. Despite of the need for a second surgery for plate removal, this technique restores functional elbow range of motion with the added advantage of minimization of postoperative restrictions as well as rate of revision surgeries in comparison to elbow replacement.

## Data Availability

All data and material of the cases are available.

## Abbreviations

AO/OTA: Association for Osteosynthesis – Orthopaedic Trauma Association
DHH: Distal humeral hemiarthroplasty
LC-DCP: Limited contact – dynamic compression plate
MEPS: Mayo Elbow Performance Score
ORIF: Open reduction internal fixation
Q-DASH: Quick Disabilities of the Arm, Shoulder and Hand
ROM: Range of motion
TEA: Total elbow arthroplasty
